# GROUP-BASED TRAJECTORY MODELING OF NURSING HOME RESIDENT PAIN SCORES

**DOI:** 10.1093/geroni/igac059.357

**Published:** 2022-12-20

**Authors:** Connie Cole, Susan Hickman, Justin Blackburn, Janet Carpenter, Chen Chen

**Affiliations:** University of Colorado Anschutz Medical Campus, Kendallville, Indiana, United States; Indiana University School of Nursing, Indianapolis, Indiana, United States; IU Richard M. Fairbanks School of Public Health at IUPUI, Indianapolis, Indiana, United States; Indiana University, Indianapolis, Indiana, United States; Indiana University, Indianapolis, Indiana, United States

## Abstract

Up to 80% of older adults living in a nursing home (NH) experience pain and up to 32% have substantial pain. Pain in NH residents is associated with poor quality of life, higher likelihood of depression, and decreased life satisfaction. Pain in NH residents has often been studied using a cross-sectional approach, which fails to consider the temporal nature of pain. Therefore, the purpose of this analysis was to identify and characterize clinically meaningful, dynamic pain trajectories in NH residents using data from the Minimum Data Set. A retrospective longitudinal analysis was conducted using group-based trajectory modeling with pain scores from admission to discharge or a maximum of 28 assessments. We identified four distinct trajectories: 1) consistent pain absence (48.9%), 2) decreasing-increasing pain presence (21.8%), 3) increasing-decreasing pain presence (15.3%), and 4) persistent pain presence (14.0%). Relative to residents’ in the consistent pain absence trajectory, the likelihood of being in the persistent pain presence trajectory was more than twice as high for those living in a rural versus (AOR 2.7, CI 2.2-3.4, p<.001), over 4 times higher for those with hip fracture (AOR 4.3 CI 2.6-7.0, p<.001), nearly 3 times higher for those with a fracture other than hip (AOR 2.9, CI 2.0-4.1, p<.001), and almost twice as high for those with contracture (AOR 1.7, CI 1.4-2.1, p<.001). Using residents’ characteristics associated with persistent pain such as hip fracture or contracture may improve care planning based on early identification or risk stratification and can improve mitigation of persistent pain.

